# Knowledge, Awareness, Perceptions, and Use of Emergency Contraceptives among Survivors of Intimate Partner Violence

**DOI:** 10.1155/2009/625465

**Published:** 2009-10-12

**Authors:** Kathleen J. Wilder, Jeane-Marie Guise, Nancy A. Perrin, Ginger C. Hanson, Rebecca Hernandez, Nancy Glass

**Affiliations:** ^1^Department of OB/GYN, Northern Navajo Medical Center, Indian Health Service, Shiprock, NM 87420, USA; ^2^Department of OB/GYN, Oregon Health and Sciences University School of Medicine, Portland, OR 97239, USA; ^3^Center for Health Research, Kaiser Permanente Northwest, Portland, OR 97227, USA; ^4^Center for Intercultural Teaching and Learning, School of Nursing, Goshen College 1700 S. Main Street Goshen, IN 46526, USA; ^5^Johns Hopkins Center for Global Health, Johns Hopkins University School of Nursing, 525 North Wolfe Street, Rm 433, Baltimore, MD 21205, USA

## Abstract

The study examines emergency contraception (EC) knowledge, awareness, perceptions, and prior use and identifies predictors of EC use among a sample of survivors of intimate partner violence (IPV). The majority (66.2%) of 154 survivors at risk of pregnancy reported EC awareness, only 15.3% reported prior EC use. Logistic regression identified perceived abusive intimate partner approval (OR = 2.25; 95% CI = 1.15–4.41) and lack of moral/religious objections (OR = 12.83; 95% CI = 5.48–30.03) as the strongest predictors of EC use. Health care provider interventions acknowledging barriers to EC use, such as partner approval, and education that improves awareness of and knowledge about EC, may have the impact of empowering survivors in their reproductive choices, reducing unwanted pregnancies.

## 1. Introduction

One and a half million women are physically assaulted by an intimate or exintimate partner every year in the United States [1]. Studies have documented a higher rate of intimate partner violence (IPV) among women with unplanned and unwanted pregnancies both in North America and low-income countries [2]. Women reporting a history of physical and/or sexual IPV are less likely to use a method of contraception compared to women not reporting IPV [3, 4]. Qualitative data derived from focus groups suggest that this lack of contraceptive use is due to a lack of negotiating power in the relationship, as contraception (use or nonuse) serves as a method of control for men over women in violent relationships [5]. Furthermore, these focus group data reveal that women in abusive relationships prefer contraceptive methods that cannot be found and destroyed by the abusive partner or methods that cannot be recognized by the partner, such as tubal ligation or depot-medroxyprogesterone acetate.

 Emergency contraceptive (EC) may be an acceptable and effective method of avoiding unplanned pregnancy for abused women as it can be instituted without partner knowledge. While there is a paucity of research about abused women's knowledge, awareness, perception and EC use, population-based studies have found moral or religious concerns to be barriers [6–8].

 Prior studies have identified racial disparities in EC awareness and knowledge, demonstrating lower rates of awareness and knowledge among Latinos, Blacks, and Asians when compared to nonHispanic whites [7, 9] and lower rates among Spanish speaking Latinas compared to English speaking Latinas [8]. Knowledge and awareness are important predictors of EC use. A study of Swedish women found that a knowledge and awareness campaign was related to increased awareness and knowledge of EC, higher usage of EC, and lower abortion rates [10]. However, in two studies, race and ethnicity were not predictors of EC use [7, 8]. Instead, attitudes and beliefs regarding mechanism of EC action were found to be more predictive of EC use. We do not know how race and ethnicity may intersect with a history of IPV to predict EC use.

 The objective of the study is to examine emergency contraception (EC) knowledge, awareness, perceptions, and prior use and identify predictors of EC use among a sample of survivors of intimate partner violence.

## 2. Materials and Methods

The Women's Sexual Health Survey (WSHS) was administered through a larger National Institutes of Health/National Institute for Nursing Research (NIH/NINR) funded study intended to develop a workplace intervention to reduce health disparities for immigrant and US-born low-income employed survivors of IPV. The WSHS was administered between June 2005 and May 2006 via face-to-face interviews to 207 female survivors of IPV living in Oregon. Adult women (18 years of age or older) were eligible to participate in the study if they spoke English or Spanish, and had been physically or sexually abused by an intimate or exintimate partner in the past year. We restricted our analyses to women less than 50 years of age and those who had not had a tubal ligation or hysterectomy (*N* = 154).

 In-person interviews were conducted at safe and convenient times and locations determined by the participants. Participants were recruited through community-based organizations (e.g., head start, parenting programs, and domestic violence services programs), housing communities, and faith-based organizations with the expert collaboration of promotoras (community health workers) and informed that refusal to participate had no effect on their participation in any program services. Institutional review board approval was granted by Oregon Health & Sciences University (IRB number 0497) and Johns Hopkins Medical Institutions (IRB number 4113).

 The WSHS is a 24-item instrument and contains a mixture of open and closed ended items. Most of the items were drawn from previously published studies of EC awareness, knowledge, and perceptions, which demonstrated their predictive validity [7–9, 11]. Additional items were developed based on the investigators clinical experience with victims of IPV. The WSHS was developed to assess history of pregnancy, sexual activity and contraception use and measure women's awareness, knowledge, perceptions and EC use. A multidisciplinary workgroup, formed from the study team and including Latina cultural consultants, provided feedback, edits, and Spanish translation/back-translation during WSHS development. The instrument was pretested with community-based advocates and promotoras (community health workers), and their feedback, suggestions and edits were incorporated in the final version of the instrument, which was administered in a face-to-face interview.

 To measure EC awareness, two questions were asked, “If a woman has just had sex and thinks she might become pregnant, is there anything she can do in the next few days to prevent pregnancy?” and “have you ever heard of morning-after pills, also called emergency contraceptive pills?” Women who were aware of EC were also asked: (1) how effective is EC? and (2) how safe is EC? (dichotomized from a four point scale to very or somewhat safe versus very or somewhat unsafe).

 To measure EC knowledge women were asked to respond to four questions: if a prescription is required to obtain EC, when EC must be taken to be most effective after unprotected sex, if EC can cause an abortion, and if it can cause problems getting pregnant in the future (At the time of this survey, a prescription was still required to obtain emergency contraception from a pharmacist in most states, including Oregon.) In order to understand the spectrum of awareness and knowledge, we created a trichotomous variable (e.g., aware and knowledgeable, aware but not knowledgeable, neither aware nor knowledgeable). Women who were neither aware nor knowledgeable of EC (answered negatively to the two awareness questions and incorrectly to the four knowledge questions) were scored 0. Those women who were aware (i.e., answered positively to the two awareness questions) and also correctly answered at least of 2 of the 4 questions on EC knowledge (i.e., prescription required, correct timing after unprotected sex, does not cause abortion, does not cause problems with pregnancy in the future) were scored 1 (low knowledge) and those who correctly answered 3 or more of the 4 questions were scored 2 (high knowledge).

 We also examined women's perceptions about EC use. For example, women's perception about their partner's approval of EC use was measured on a 4-point scale (i.e., partner strongly disapproves, somewhat disapproves, somewhat approves, strongly approves) and was dichotomized for the analysis into somewhat or strongly disapprove versus somewhat or strongly approve.

 EC use was measured according to women's responses to, “if there were pills that you could take after unprotected sex to prevent pregnancy, how likely would you be to ever use them?” (coded as very or somewhat likely versus somewhat or very unlikely for analysis). All women were also asked about barriers to EC use: (1) not knowing where to get EC, (2) lack of transportation, (3) lack of money to buy EC, (4) could not tolerate the side effects, and/or (5) moral or religious objections against using EC.

 Data were analyzed using Statistical Package of the Social Sciences 15.0. The first set of analyses compared women with awareness/knowledge of EC to those without, with use of EC as the dependent variable. The relationship between each predictor and the dependent variable was tested using logistic regression. Those with significant relationships were simultaneously entered into a final logistic regression to assess independent predictors of EC use. Finally, we conducted a subanalysis among women who were aware of EC to further understand predictors for EC use in this population.

## 3. Results

The eligible sample of IPV survivors at risk for pregnancy included 154 women with a mean age of 32.5 years (SD = 7.3, range = 18 to 47) and 47.4% Latina. Table 1 describes the demographics, reproductive and contraceptive history of the sample. All women interviewed had sexual intercourse and 86.4% had been pregnant at least once. Nearly half (48.1%) of the sample reported forced sex by an intimate or exintimate partner in the past year. Over half (59.7%) had a history of a prior unplanned pregnancy and 23.4% had at some time received help to end a pregnancy. Two-thirds of the sample reported using some method of birth control at prior consensual intercourse (69.5%) and 15.3% reported prior EC use.

Table 2 describes the awareness/knowledge variable distribution as well as the barriers to EC use for the sample of 154 women. Sixty-six percent of women had some awareness and knowledge of EC. Among those with awareness/knowledge of EC (Awareness/Knowledge score of 1 or 2), the vast majority (87.3%) perceived EC to be effective and 61.8% perceived it to be safe. However, the majority (62.7%) also thought that if a woman is already pregnant, that EC will cause an abortion and almost half (49.0%) thought taking EC may cause problems getting pregnant later.

 The logistic regression revealed that none of the demographic variables (i.e., education, married or partnered, age, or race/ethnicity) or experiencing forced sex by an intimate or exintimate partner were significantly related to EC use. Abused women reported important barriers to EC use such as not knowing where to get EC (26.6%), lack of transportation (17.5%), not having money to buy EC (37.7%), and not able to tolerate the side effects (27.3%). However, these barriers were not significantly related to EC use in this sample.

 Further analysis found that abused women who reported no moral/religious objections to EC use were nearly 13 times more likely to use EC than those abused women who had moral/religious objections to EC use (OR = 12.83; 95% CI = 5.48–30.03). Intimate partner's approval was also a significant predictor of EC use, women who perceived that their abusive partner would approve of EC were over twice as likely to use EC (OR = 2.25; 95% CI = 1.15–4.41). The awareness/knowledge variable was a significant predictor for EC use, with women with more knowledge being more likely to use EC (OR = 1.67; 95% CI = 1.02–2.74).

 When all three significant variables (moral/religious objections, partner approval, awareness/knowledge) were included in the model simultaneously, moral/religious objections to EC use and partner's approval of EC use remained significant, EC awareness/knowledge was no longer significant (Table 3).

 We then conducted the analysis with a subsample of abused women, the 102 women (66.2% of the sample) who reported awareness/knowledge of EC. Women who reported that EC does not cause abortions, were 3 times more likely to use EC than those who reported EC caused abortions (OR = 3.05; 95% CI = 1.20–7.75). Women who reported that EC use does not cause problems with becoming pregnant later were 2.4 times more likely to use EC than those who reported EC will cause problems (OR = 2.43; 95% CI = 1.07–5.54). As shown in Table 4, among women who reported being aware/knowledgeable of EC, knowledge that EC does not cause an abortion was the strongest predictor of EC use.

## 4. Discussion

To our knowledge, this is one of the first studies to examine awareness and knowledge, perceptions, and use of emergency contraception in a sample of IPV survivors. Compared to the general population, this sample of women reports a higher incidence of forced sex, unintended pregnancy, and prior EC use, but a lower incidence of terminated pregnancy [12], confirming this to be a vulnerable population for unintended and potentially unwanted pregnancies. Also notable is the lack of association between race or ethnicity and EC use, implying that EC is an acceptable option to IPV survivors regardless of race or ethnicity. Similarly, Thorburn et al. [13], found acceptance of EC in a qualitative study among Mexican women living along the US-Mexico border who had been raped or sexually abused by an intimate partner or other. EC access and use could potentially provide women in abusive relationships an opportunity to take some control over their reproductive choices even though they may not have control over other aspects of their relationships.

 In order to understand how our sample of IPV survivors may be different from other samples where IPV history is not known, we examined literature. Abbott et al. [11] published a study of EC knowledge, attitudes, and use among a sample of 158 women presenting to an inner-city emergency department (ED), Romo et al. [8] studied a population of 297 Latino women receiving care in university reproductive health clinics, and Jackson et al. [7] studied a diverse sample of 371 post-partum women, 72% of whom were Latina. Our study findings among a sample of IPV survivors demonstrate similar awareness and EC use to other populations in the published literature. However, our sample has lower rates of knowledge (as measured by questions on the following: prescription is required to obtain EC, when EC must be taken to be most effective after unprotected sex, if EC can cause an abortion, and if it can cause problems getting pregnant in the future). Importantly, the length of time between the published research and our study could account for an increase in EC awareness among the sample, as EC has been the subject of much discussion and debate in the popular media, but poorer knowledge among our sample is a concern as they are at high-risk for forced sex and an unplanned pregnancy.

 Additionally, it is unknown how the physical and psychological abuse in women's lives as well as poverty and lack of health insurance may limit their access to accurate EC information. The regression models demonstrated that moral/religious objections to EC use to prevent pregnancy after unprotected sex and women's perception of partner's approval of EC use were significant predictors of EC use. The association with moral or religious objection to taking EC to prevent pregnancy has been identified in other studies [7, 8]. It is also well known that a partner's approval plays an important role in family planning for contraception and induced abortion. However, this study is unique in its identification of the importance of woman's perception of abusive partner's approval on their EC use. Given the issue of control in abusive relationships, the partner's approval, or perceived approval, is an important barrier to EC use in this group. Future analyses should examine what aspects of the relationship may be related to this perception. For example, do the women report being less likely to use EC, because they know they will have difficulty accessing the medication without the abuser's assistance (monetary support, transportation), or because they fear the repercussions from the abuser, such as violence or accusations of infidelity if the abusive partner were to find out she was taking EC.

 Previous research has indicated that fear of side effects [14–16] and economic reasons [17] are common barriers to EC use. While we found that a notable number of women reported these concerns. No evidence was found that these were significant factors in women's future intentions to use EC. One possible explanation for this discrepancy is that other studies have tended to use qualitative or descriptive methodology while we relied on statistical techniques. Based on our descriptive findings alone we might have come to the same conclusions. Another explanation could be the differences in study populations. Previous studies have largely been conducted with young women, this study focused on women experiencing IPV. While women experiencing IPV may have similar concerns about EC, these concerns might be overshadowed by other issues unique to their situation.

 The study has important limitations. First, the study consists of a cross-sectional survey with a sample of low-income abused women, so generalizability is limited. However, we did successfully recruit a racially and ethnically diverse sample of abused women that provides increased understanding related to awareness, knowledge, perceptions, and EC use among Latina and nonLatina women. Additionally, without a comparison group of nonabused women, we cannot know if the partner's approval or disapproval of EC use would influence nonabused women's EC use or if it is unique to abused women.

## 5. Conclusions

The predictors of EC use among women who were aware of EC demonstrate that awareness of EC is not synonymous with knowledge. The perception that EC is an abortifacient and may cause future fertility problems serve as barriers to EC use among this sample of IPV survivors. Thus, providing accurate information about how EC works is important for this population is critical.

 We have three recommendations for clinical and social service providers based on our findings that forced sex is common in abusive relationships, risk of unwanted pregnancy is high, and that EC use among IPV survivors is influenced by moral/religious objections, inaccurate knowledge, and perceptions of the abuser's approval. First, since the healthcare and contraceptive needs of IPV survivors are unique, healthcare providers may benefit from routinely assessing for physical and sexual IPV during clinical encounters. There are several well-validated short assessment tools such as the 4-item Abuse Assessment Screen [18] that includes a question on forced sex and can be used to assist providers in diverse health care settings to routinely assess for IPV.

 Second, comprehensive safety planning for women in abusive relationships needs to include reproductive health education. Health care or social services providers who are already assisting women in abusive relationships with healthcare, housing, or obtaining restraining orders should also include an assessment of the woman's awareness and knowledge of contraception. As in the general population, it is important to inform women that EC is less effective than other methods of birth control and may be better suited as an occasional back up form of birth control [19]. However, is should be noted that the controlling nature of these women's relationships may present a barrier to other forms of birth control, making EC a more attractive option. Thus it is important to include a discussion of the existence, accessibility, and safety of EC to prevent an unplanned pregnancy and knowledge regarding its mechanism of action (i.e., EC is not an abortifacient). This educational information should be provided without regard to race or ethnicity but within the context of the woman's religious and moral objections as well as her perception of the abusive partner's approval of EC use. If the woman perceives the partner will not approve, the health care provider or other service provider should discuss strategies for safely accessing and using EC without their partner's knowledge.

 Lastly, lay community members (e.g., Promotoras de Salud) can be trained to address basic reproductive health care and be equipped with appropriate referral resources. Many of the women in our sample were Latinas who are not of US origin and who are monolingual Spanish. Reproductive health resources need to be available and lay community health workers are one way to reach women who may not access traditional health care or insurance.

## Figures and Tables

**Table 1:** Demographic and reproductive health characteristics of the sample.

	Total sample (*n* = 154)
Demographic Characteristics	

Average Age (sd)	32.5 (7.3)
Race/Ethnicity	
* *White	31.2%
* *Black	7.8%
* *Asian	1.3%
* *American Indian	5.8%
* *Pacific Islander	1.3%
* *Hispanic	47.4%
* *Other	1.3%
* *Multi-racial	3.9%
Monolingual Spanish Speaking	27.2%
Average Years of Education (sd)	10.9 (3.2)
Married or living w/partner	44.2%
Employed Outside the Home	61.7%

Reproductive Health Characteristics	

Ever pregnant	86.4%
Ever had unplanned pregnancy	59.7%
Ever received help to end a pregnancy	23.4%
History of forced sex in last year	48.1%
Prior EC use	15.3%

**Table 2:** Emergency contraception awareness, knowledge, perceptions, and likelihood of use in the future for the sample.

EC Awareness/Knowledge*	Total Sample (*n* = 154)
* *Unaware	33.8%
* *Low Knowledge	51.3%
* *High Knowledge	14.9%
Likelihood of future EC use	57.0%
* *Reasons for not using EC in the future	
* *Believe partner would not approve	42.2%
* *Donot know where to get it	26.6%
* *Lack of transportation	17.5%
* *Lack of money to buy it	37.7%
* *Believe she could not tolerate side effects	27.3%
* *Has moral or religious objections	31.8%
Knowledge among those aware of EC	*N* = 102
* *If a woman is already pregnant EC will cause an abortion	62.7%
* *EC may cause problems with becoming pregnant later	49.0%
Perceptions among those aware of EC	
* *Believe that EC is effective	87.3%
* *Believe that EC is safe	61.8%

*“Those unaware answered no or donot know to the questions, “if a woman has just had sex and thinks that she might become pregnant, is there anything she can do in the next few days to prevent pregnancy?” and, “have you ever heard of morning-after pills, also called emergency contraceptive pills?” Low knowledge and high knowledge indicate the woman was aware of EC and correctly answered at least of 2 or 3 of the 4 questions on EC knowledge (i.e., prescription required, correct timing after unprotected sex, does not cause abortion, and does not cause problems with pregnancy in the future), respectively.

**Table 3:** Logistic regression predicting likelihood to use emergency contraception.

	OR (95% CI)
No moral or religious objections	13.66 (5.57–33.48)
Partner approves	2.56 (1.13–5.77)
Level of knowledge/awareness	1.44 (.792–2.61)

**Table 4:** Logistic regression predicting likelihood to use emergency contraception for IPV survivors aware of emergency contraception.

	OR (95% CI)
Will not cause an abortion	2.64 (1.01–6.86)
Will not cause problems becoming pregnant later	1.94 (.82–4.59)
